# Professional Development to Improve Responsible Beverage Service Training: Formative Research Results and Protocol for a Randomized Controlled Trial

**DOI:** 10.2196/49680

**Published:** 2024-01-24

**Authors:** W Gill Woodall, David Buller, Robert Saltz, Lila Martinez

**Affiliations:** 1 Klein Buendel, Inc Golden, CO United States; 2 Prevention Research Center Pacific Institute for Research and Evaluation Berkeley, CA United States

**Keywords:** alcohol, driving while intoxicated, responsible beverage service, training, prevention, professional development, social media

## Abstract

**Background:**

Improved interventions are needed to reduce the rate of driving while intoxicated. Responsible beverage service (RBS) training has reduced service to intoxicated patrons in licensed premises in several studies. Its efficacy might be improved by increasing the proper application and continued use of RBS with a professional development program in the 3 to 5 years between the required RBS retraining.

**Objective:**

This study aims to develop and evaluate a professional development component for an RBS training that aims to improve the effectiveness of the web-based training alone.

**Methods:**

In a 2-phase project, we are creating a professional development component for alcohol servers after completing an RBS training. The first phase involved formative research on the feasibility, acceptability, and potential effectiveness of components. Semistructured interviews with owners and managers of licensed establishments and focus groups and a survey with alcohol servers in New Mexico and Washington State examined support for RBS and the need for ongoing professional development to support RBS. A prototype of a professional development component, *WayToServe Plus*, was produced for delivery in social media posts on advanced RBS skills, support from experienced servers, professionalism, and basic management training. The prototype was evaluated in a usability survey and a field pilot study with alcohol servers in California, New Mexico, and Washington State. The second phase of the project will include full production of the professional development component. It will be delivered in Facebook private groups over 12 months and evaluated with a sample of licensed premises (ie, bars and restaurants) in California, New Mexico, and Washington State (n=180) in a 2-group randomized field trial (*WayToServe* training only vs *WayToServe* training and *WayToServe Plus*). Licensed establishments will be assessed for refusal of sales to apparently intoxicated pseudopatrons at baseline and 12 months after the intervention commences.

**Results:**

Although owners and managers (n=10) and alcohol servers (n=43) were favorable toward RBS, they endorsed the need for ongoing support for RBS for servers and identified topics of interest. A prototype with 50 posts was successfully created. Servers felt that it was highly usable and appropriate for themselves and the premises in the usability survey (n=20) and field pilot test (n=110), with 85% (17/20) and 78% (46/59), respectively, saying they would use it. Servers receiving the professional development component had higher self-efficacy (*d*=0.30) and response efficacy (*d*=0.38) for RBS compared with untreated controls.

**Conclusions:**

Owners, managers, and servers believed that an ongoing professional development component on RBS would benefit servers and licensed premises. Servers were interested in using such a program, a large majority engaged with the prototype, and servers receiving it improved on theoretic mediators of RBS. Thus, the professional development component may improve RBS training.

**Trial Registration:**

ClinicalTrials.gov NCT05779774; http://tinyurl.com/4mw6d2vk

**International Registered Report Identifier (IRRID):**

DERR1-10.2196/49680

## Introduction

### Background

Driving while intoxicated (DWI) is one of the most preventable public health risks in the United States. However, from 2019 to 2020, there was an increase of 14.3% in DWI deaths, after remaining largely stable from 2015 to 2019 [1]. Although new policies and interventions are needed to reduce the consequences of DWI, gains are possible by increasing the efficacy of existing interventions. Responsible beverage service (RBS) training [2-4] has been effective in some cases [5], but methods of boosting its efficacy are needed. The goal of this research is to develop and evaluate a professional development component for a web-based RBS training program that aims to improve the effectiveness of web-based training. The professional development component will provide ongoing information and instruction in advanced RBS techniques and emphasize professionalism in the hospitality industry. It will be continuously available and easily accessible to alcohol servers via social media after completing the web-based training.

Marketplace approaches to prevent alcohol service that results in intoxication or restrict access to alcohol by persons already intoxicated are an alternative policy approach to deterrence of driving by drinkers considered impaired to decrease DWI [6]. Most US states have laws prohibiting sales of alcohol to visibly intoxicated customers [7]. A complementary intervention is RBS training, which aims to instruct servers on how to prevent intoxication by teaching drink counting techniques, ways to recognize signs of intoxication, and strategies to refuse alcohol sales. This environmental intervention aims to decrease opportunities for risky behavior [8], consistent with harm reduction in a nurturing environment perspective [9]. It is a targeted restriction on alcohol accessibility at the times and places where risk is greatest that does not depend on decision-making by persons considered alcohol impaired, can be applied to all alcohol sales premises, does not depend on house policies of licensees to refuse sales, and reduces intoxicated customers’ ability to *shop around* to find premises that will serve.

Research on RBS training presents a mixed picture [5]. Although some studies have failed to show effectiveness [10], reviews in 2000 and 2001 concluded that RBS training can prevent alcohol overservice [11] with strong management support [12]. Recent studies have found RBS training to be associated with increases in refusals of service to apparently intoxicated customers. In addition it was related to decreases in blood alcohol concentration and calls to emergency services [13]. RBS training combined with enforcement reduced alcohol overservice and violent assaults in a trial in Sweden [14-16] but not in Norway [17]. In addition, lower levels of motor vehicle crashes with a high percentage of alcohol involvement were observed in a mandatory RBS training state [18], and another analysis found that states with RBS laws had a reduced number of underage drinking driver fatality crash ratios [19]. Data on self-reported DWI have been mixed, with one study showing no association [20] and another finding a decrease in reported DWI with RBS training [13]. Continued research on RBS training [21] is warranted because (1) positive outcomes have been reported [5,16,22,23]; (2) methodological problems limit existing evidence (eg, lack of randomized trials, clear outcome variables, training fidelity data, and effect size reporting) [5,11,12]; and (3) data are limited on web-based training that can improve training engagement, fidelity, and quality, compared with in-person training [5]. Our team showed that a web-based RBS training program, named *WayToServe*, was effective in premises serving alcohol for onsite consumption (ie, bars and restaurants) [24].

RBS training laws are highly variable across US states [25]. It is legally mandated in 25 US states and incentivized in some fashion in a number of other states [26]. Most states that require training have long periods of 3 to 5 years between required retraining. Consequently, the proper application, monitoring, and continued use of the RBS techniques falls on the shoulders of premises management, so it is not surprising that management commitment to RBS can affect servers’ adherence to RBS methods [5,12]. Developing ways to support RBS techniques after training may counter the management’s ineffective or limited support for, disinterest in, or outright resistance to RBS.

### Objectives

The goal of this study is to develop and evaluate a follow-on professional development component to increase the efficacy of our web-based RBS training, *WayToServe*. Continuing professional development is a widespread practice across a variety of fields including accounting, social work, and medicine [27]. Typically, it focuses on improving knowledge, skills, and performance to help employees stay up-to-date on industry developments, develop and maintain job capabilities, convey professional values and norms, and create communities of practice [27-32]. Although often focused on *high-skill* professional workers (eg, nurses, physicians, lawyers, and architects) [27], the training and certification of community members has improved professionalism [33] and, along with in-service contact, has boosted the success of community prevention programs [34,35]. Vocational education and lifelong learning play essential roles in the hospitality industry. They offer both general knowledge and skills such as communication and customer service as well as job-related knowledge such as understanding laws related to serving alcohol and the ability to recognize signs of intoxication [32,36,37]. Alcohol servers trained in RBS practices should benefit from ongoing professional development by (1) motivating them to implement RBS skills in the face of common barriers, such as pressure to sell, low management support, and customers’ attempts to continue being served; (2) receiving support for RBS from a *community* of alcohol servers, especially for servers who work in small or unsupportive premises; and (3) preventing the degradation of RBS skills over time.

## Methods

### Overview

This study is being conducted in 2 phases to create an effective professional development component for alcohol servers who completed state-approved RBS training. The first phase aimed to determine whether a professional development component delivered over social media was feasible, acceptable, and potentially effective for alcohol premises management and servers. The second phase will involve production of the professional development component and testing its efficacy in a randomized controlled trial (RCT).

### Phase 1: Formative Research

#### Overview

A formative research phase in 2022 used both qualitative and quantitative techniques to provide a detailed picture of RBS training and its gaps as well as the feasibility and potential effectiveness of adding a professional development component. It included semistructured interviews with premises managers, focus groups and a survey with alcohol servers, development of a prototype of the professional development component, and usability testing and a field pilot study evaluating the prototype.

#### Semistructured Interviews With Alcohol Premises Owners and Managers

Owners and managers (n=10) of onsite alcohol premises in New Mexico and Washington State participated in semistructured interviews. Premises had to hold an active state alcohol license and be a bar or restaurant that served alcohol. Managers discussed RBS policies at their premises, support for RBS methods, perceived importance of RBS, quality of RBS methods implemented by their servers, and need for ongoing training and support for RBS among their servers. They also suggested content for a professional development component. The transcripts were coded to identify themes.

#### Focus Groups and Survey With Alcohol Servers

Alcohol servers working in onsite alcohol sales premises in New Mexico and Washington State were recruited from the roster of *WayToServe* trainees to provide input on experience with RBS methods and interest in ongoing professional development related to RBS methods. To be included, servers had to be aged ≥19 years (by state regulation), serve alcoholic beverages at a licensed premises, have completed the *WayToServe* RBS training, and be proficient in English. Initially, alcohol servers (n=19) were recruited to web-based focus groups; however, when participation lagged, servers (n=24) were recruited instead to complete a web survey. In both the focus groups and survey, servers were asked about their experience with, confidence in, and barriers to RBS methods; support from management and other alcohol servers for RBS; and experience refusing service to customers. They also indicated their interest in and potential utility of receiving ongoing information and activities from *WayToServe* to keep up-to-date and be capable of using RBS methods via a Facebook group. Servers reported whether they were willing to share their RBS experiences or provide feedback on RBS actions with other servers. Focus group transcripts were coded for themes for each question. The survey responses were summarized using descriptive statistics.

#### Production of a Prototype Professional Development Component

A prototype of the professional development component was produced by the project staff and media developers. Named *WayToServe Plus*, it comprised a series of 50 social media posts. The goal of the messages was to improve servers’ professionalism by (1) increasing the confidence and motivation of RBS-trained servers to implement RBS methods, with attention to ways of overcoming common barriers; (2) creating a professional community of servers that supports one another in implementing RBS actions and serves as a resource for advice and strategies to implement RBS (eg, tips and tricks) by encouraging servers to share their personal experiences through comments and posts; and (3) preventing the deterioration of RBS skills and motivation over time by providing refresher instruction. Posts contained text, graphics, web-based learning activities, and videos demonstrating RBS techniques in 4 topic areas derived from the results of the manager interviews and alcohol server focus groups and surveys: advanced RBS skills training (ID checking, cannabis and alcohol, drink counting, and home delivery), experienced servers supporting new servers (eg, tips and tricks to apply RBS and sharing stories on RBS experiences), professionalism (safety and security, security personnel, and handling disruptive customers), and basic management training (content and development of house RBS policies and best practices for RBS). Instructional goals included improving the application of RBS information and skills in realistic settings and circumstances that servers have encountered in their jobs. *WayToServe Plus* was consistent with the transformative approach to continuing professional development by Kennedy [38], combining the transmission of information, skills, and norms and providing coaching or mentoring by striving to create a community of practice among servers with varying levels of experience. Messages in the posts were guided by principles of diffusion of innovation theory (eg, compatibility, simplicity, trialability, and observability) [39] and social cognitive theory (ie, modeling) [40] and written to be relatable, positive, and entertaining. A total of 14 short videos were produced using the TikTok video authoring platform. An interactive quiz activity was taken from the *WayToServe* training and linked to a social media post. *WayToServe Plus* was authored in English because most servers had elected to complete *WayToServe* training in English.

#### Evaluation of Prototype Professional Development Component

The prototype *WayToServe Plus* component was evaluated for usability, feasibility, acceptability, and engagement through a survey and a field pilot test with alcohol servers recruited from the roster of *WayToServe* trainees.

##### Usability Testing

Alcohol servers (n=20) who met the aforementioned inclusion criteria (refer to the *Focus Groups and Survey With Alcohol Servers* section) completed a web survey on prototype acceptability, feasibility, and utility (10 in New Mexico and 10 in Washington State). Ten usability testers can identify 95% of problems [41-43]. In the survey, servers were provided with a description of the *WayToServe Plus* component. Each server was presented with 3 posts and 1 video randomly selected from posts in the prototype. They were then asked to evaluate these items based on their appropriateness for themselves and licensed establishments, their acceptability, and usefulness, using 5-point Likert scales. Servers indicated if they would read or view the post or video, react to it (eg, like, sad, and angry), comment on it, and share it. A description of the interactive activities was provided and rated based on these measures. Next, servers evaluated the *WayToServe Plus* concept on the validated System Usability Scale (SUS) [44-46]. The 10 items were combined using standard techniques, with a score of ≥68 indicating adequate usability. In addition, a single item assessed user-friendliness (1=worst imaginable and 7=best imaginable). Finally, servers indicated whether they would be interested in getting the ongoing information and activities from *WayToServe Plus*, topics that would be of interest to them, and potential reasons for not using it.

##### Field Pilot Test

A sample of 59 alcohol servers (5 in California, 21 in New Mexico, and 33 in Washington State) participated in a 1-month field pilot test of the prototype, meeting the same inclusion criteria as the focus group and usability testing participants. The study involved a nonrandomized posttest-only 2-group design, in which the treatment group had 2 levels: prototype *WayToServe Plus* program versus no treatment. In the intervention group, 59 servers were recruited and joined a Facebook private group on a rolling basis over 8 weeks. Staff posted prototype *WayToServe Plus* posts (1 per day, Monday to Friday) for the 8-week period. Approximately 24 posts were posted to the private group during any 4-week period in the intervention period. The posts were only viewable to members of the private group and could not be shared outside the private group. Outcomes were assessed in 2 ways at 4 weeks after enrollment. First, servers’ engagement with *WayToServe Plus* was measured by recording the number of times posts were viewed, reacted to (eg, liked), and commented on by servers. Second, servers completed a web-based posttest, assessing the prototype on appropriateness, acceptability, and utility for servers and premises and usability on the SUS [44-46] and whether the tone of the prototype aligned with their licensed establishment’s atmosphere, using scales similar to those used in the usability survey. Perceived self-efficacy and response efficacy for maintaining community safety by using RBS methods (5-point Likert scales) were measured as proxy outcomes of the effectiveness of the prototype program (ie, dependent variables). Willingness to use the *WayToServe Plus* program in the future and job and demographic characteristics were also measured. The respondents suggested ways to improve the prototype and make it more engaging. A second group of 51 servers was recruited to serve as an untreated control group and completed only a posttest web survey, with the primary purpose being to assess their perceived self-efficacy and response efficacy of RBS methods and compare them with ratings provided by servers who received the prototype.

### Phase 2: RCT Protocol for Evaluating the Professional Development Component

The *WayToServe Plus* professional development component will be fully produced and evaluated for effectiveness in an RCT.

#### Production and Implementation of WayToServe Plus Component

A 12-month version of the *WayToServe Plus* professional development component will be created for evaluation in the trial. Content and format will be developed according to instructional goals, principles from diffusion of innovation theory and social cognitive theory, and insights derived from the formative research findings in phase 1. Posts will contain text, infographics, short videos, and interactive activities based on the *WayToServe* RBS training. Features to elicit user-generated content will be included in posts, such as posing a common situation and asking, for example, RBS strategies; providing polls about RBS methods; and soliciting stories, tips, and tricks from experienced servers for applying RBS. These posts are intended to create sense of community among alcohol servers. An agile iterative production process will be used to author the posts [47]. Approximately 2 months of posts will be prepared before launching the intervention; additional posts will be developed during the intervention, adjusting them for season, current events, and reactions and comments from servers to prior posts.

*WayToServe Plus* will be administered by a staff member who serves as a community manager. The manager will post 4 posts per week, Monday through Friday, for 12 months (approximately 208 posts in total). In addition, posts selected from the usual-and-customary *WayToServe* Facebook page will be posted once per week. Alcohol servers can comment on and react to posts but cannot share them on their own feed. Orientation to private groups will be self-explanatory. The community manager will stress respect for others; monitor comments; and correct inappropriate, unfavorable, or bullying comments or misinformation [48]. Servers will be compensated US $50 for joining the *WayToServe Plus* Facebook private group.

#### Randomized Trial Design

The *WayToServe Plus* professional development component will be evaluated with a sample of 180 establishments licensed for sale of alcohol for onsite consumption (ie, liquor by the drink) and their alcohol servers. Premises will be enrolled in a 2-group randomized field trial (*WayToServe* training only [comparison control] vs *WayToServe* training plus *WayToServe Plus* [intervention]) with 2 assessment rounds (baseline and posttest [12 months after intervention commences]), yielding a 2 (treatment)×2 (assessment time) factorial design. Using a custom-written program, the project biostatistician will randomly assign half of the premises (90/180, 50%) to *WayToServe* training plus *WayToServe Plus*, stratified by state (ie, independent variables). The remaining half of the premises (90/180, 50%) will receive *WayToServe* training only. All premises will be recruited to have servers complete the *WayToServe* training. Servers in the intervention group will be joined to a *WayToServe Plus* Facebook private group after training to receive the professional development component. To be added to the group, servers will friend the community manager, the manager will invite them to join the group, and servers will accept this invitation. Servers in the control group will be invited to join the usual-and-customary *WayToServe* Facebook page, which is administered by the training company. Premises will be assessed for refusal of alcohol service to visibly intoxicated patrons, the primary outcome or dependent variable and a measure of the impact of *WayToServe Plus* on actual practice [29], using a pseudointoxicated patron (PiP) protocol at baseline and at posttest. The PiP protocol presents a server with the most overt situation in which alcohol service should be refused (ie, when a patron shows clear signs of intoxication), models the behavior of patrons most at risk, and is relatively low cost. The PiP protocol has been used in thousands of alcohol premises [49], including by the research team [50-52]. PiP teams will be blinded to experimental conditions, and premises owners, managers, and alcohol servers will be blinded to PiP assessments.

#### Selection and Recruitment of Licensed Alcohol Premises

State-licensed onsite alcohol establishments (n=180) in California (n=60), New Mexico (n=60), and Washington State (n=60) were randomly selected from publicly available lists from state alcohol regulation agencies, stratified by location (metro areas [Albuquerque, San Francisco {including Oakland and San Jose}, and Seattle; n=148 premises] vs suburban towns [n=7 towns and 32 premises]). As in the formative research, they had to hold an active state license to sell alcohol and be a bar or restaurant that sold alcoholic beverages. To control travel costs in the large San Francisco and Seattle metropolitan areas, *clusters* of establishments were constructed by randomly selecting seed premises. Next, 14 additional establishments were randomly selected from within the same zip codes of the seed premises. Within each seed area, 5 to 7 establishments were selected at random for PiP visits, with the remaining premises serving as replacements for any deemed ineligible (eg, do not sell alcohol for onsite consumption) or that were closed (either permanently or during evening hours) when visited by PiP teams. In New Mexico, the Albuquerque metropolitan area was much smaller geographically, so we selected premises at random from the state lists.

After the baseline PiP assessment, project staff will contact premises management, describe participation, record the number of alcohol servers, and obtain agreement to participate in the study. Premises will be given a voucher to provide to their servers to access the *WayToServe* training and complete it within 4 weeks from registration. Severs will complete a consent form. For completing the training, servers will receive US $35 and a new server training certificate for their state. *WayToServe* will remain available to the participating premises throughout the trial, and managers will be asked to have newly hired alcohol servers complete it.

#### PiP Assessment Protocol

The primary outcome (ie, dependent variable) will be refusal of sale of alcoholic beverages to visibly intoxicated patrons assessed using a PiP protocol. Ethnically diverse male and female legal-age individuals (aged ≥ 21 years) will be hired as confederates, chosen for prior acting experience, and trained to feign intoxication when acting as buyers [50-52]. Signs of intoxication (ie, fumbling with keys or cash, swaying, slurred speech, and stumbling) indicate a high level of alcohol intoxication [53], provide a clear unambiguous choice whether to serve, and are signs that alcohol servers are trained to recognize in the *WayToServe* training and *WayToServe Plus* component. In each round (baseline and posttest), assessment will involve 2 PiP buyer visits per premises by the PiP team comprising a buyer and an observer, separated by at least 6 weeks. At each visit, observers will enter the premises before the buyer and position themselves to be able to see the buyer-server interactions. Buyers will enter the premises displaying intoxication signs and order an inexpensive beer. Both buyers and observers will record if alcohol servers agree to serve the buyers the requested alcoholic beverage. Buyers will also record if the drink was served either as requested, with reluctance, with a joke or similar remark, or with a warning that no future drinks will be served.

In addition, buyers will note the type of beverages requested, if their ID was requested, and other responses by the alcohol servers (made statements of risk, enlisted other patrons to support nonsale, offered a nonalcoholic beverage instead, offered food, provided other information [offer of taxi or safe ride, drinking facts, etc], or delayed or ignored service). Observers will record the characteristics of the establishments (state, type, number of staff and patrons, warning signs posted, and cleanliness), rate how busy the establishment is and speed of service, note if staff appear overly familiar with customers, and record the behavior of buyers (type of drink ordered, signs of intoxication displayed, and rating of obviousness of signs of intoxication). Both buyers and observers will record the servers’ job at the establishment (bartender, server, manager, bouncer, or other) and apparent sex (male, female, or do not know), Hispanic ethnicity, and race.

#### Outcome Analysis

The analysis of study outcomes will test the following hypothesis that compared with premises in *WayToServe* RBS training only group, premises assigned to receive *WayToServe* RBS training and *WayToServe Plus* will have higher rates of refusing PiP at posttest.

In our prior research, the uptake of training in alcohol establishments affected refusal rates [54], so we will test whether improvements in refusal rates are associated with uptake of the *WayToServe* training and engagement with the *WayToServe Plus* component. Training uptake will be obtained from the *WayToServe* web-based program database (ie, the number of servers registered, training modules completed, and completion of the training). Engagement with *WayToServe Plus* will be assessed by counting the number of reactions and comments on posts by servers within each premise [55]. We will not be able to count the views of posts because our sample size exceeds 250 participants; Facebook does not report views of posts in private groups with >250 users. Characteristics of alcohol establishments (type of license, type of business [bar or restaurant], how busy the premises was, and number of staff present during visit), alcohol servers interacting with PiP buyers (sex and ethnicity observed by PiP observers), and PiP buyers (sex and ethnicity) will be analyzed initially as control variables and then in subsequent models as effect modifiers of *WayToServe Plus*.

#### Interviews of Owners and Managers on WayToServe Plus Feasibility

After posttesting of establishments is completed, 18 owners and managers (6 per state) from premises in the *WayToServe* training and *WayToServe Plus* groups will be selected at random for interviews about *WayToServe Plus*, its compatibility with premises’ RBS policies and practices, helpful features, server engagement, suggested improvements, and problems or barriers (compensation=US $75). In addition, alcohol servers in these premises will be surveyed about the same issues and report their engagement with *WayToServe Plus,* whereas servers in the control premises will be surveyed about the *WayToServe* web-based training.

### Ethical Considerations

The protocols used in the formative research and randomized trial were reviewed and approved by the WCG institutional review board (#20211770). Participants read and signed an informed consent form (interviews, focus groups, and pilot field trial) or read and acknowledged a consent statement (surveys) approved by the institutional review board that described the purpose of the research, the research procedures, known risks and benefits, and the use and security of the data. Participants were informed that their participation was voluntary and that they could withdraw at any time without penalty. Participants were informed that the data collected from them in the study would be confidential and that their identity would not be disclosed in any public presentation. The participants were compensated as follows: interviews (US $75), focus group discussion (US $50), survey (US $25), usability test (US $50), and field pilot study (US $100).

## Results

### Phase 1

#### Owner and Manager Interviews About Professional Development

All owners and managers (n=10; 4 female individuals; 1 African American and 5 Hispanic White individuals) indicated that they supported RBS methods to maintain their establishment’s reputation in the community; keep the community safe; and avoid fines, disruptions, and other problems. They supported alcohol servers by addressing RBS methods in mandatory staff meetings and trainings and manager logs. They said that support for RBS was provided by experienced staff. Although most felt the RBS methods were effective at their premises, they did indicate that bartenders have many job tasks and need more help, and some establishments had more difficulty with RBS methods during summers when patrons drank longer and larger quantities of alcohol.

All owners and managers endorsed the need for ongoing training and support for RBS methods and felt that a program could help them support RBS practices. They desired topics such as checking IDs, new recreational marijuana laws, special venues (eg, music venues, wineries, and events), and communication and conflict resolution. They preferred formats such as educational memes, videos, shared experiences and tips and tricks from experienced servers, resource pages, reminders, and work group chats. Owners and managers felt that a variety of staff would benefit from ongoing training. Some of them did not feel confident addressing topics such as marijuana laws, how to handle patrons with children, and how to manage servers’ desires to sell alcoholic beverages and make money. All owners and managers would be interested in a program that provided ongoing training and support for RBS if it provided new, relevant content in engaging, easily digestible formats without a large time commitment. They were mostly or very likely to use such a program with their alcohol service staff.

#### Focus Groups and Survey of Alcohol Servers About Professional Development

##### Focus Groups

Alcohol servers participating in the focus groups (n=19) were employed in bars, restaurants, and other premises (eg, ski resort, theatre, and market) as bartenders, servers, and other staff. They had worked as servers for 2 months to 6 years.

Most servers had positive experiences applying RBS methods. They reported that owners and managers at their establishment considered RBS methods to be positive, took them seriously, and supported using them. A few servers said that they received very little support, support only from direct supervisors, and support only when they did something incorrectly. They cited management turnover and very large venues as situations that reduced support for RBS. Obstacles to RBS included customers drinking before arriving; pressure to not check IDs or provide heavier pours to regular customers or members of clubs; customers not wanting to hand over ID during COVID-19 social distancing rules; ability to check IDs from different states or military IDs; fake IDs; pressure to sell and fear of losing tips; large, busy events; and potential for negative reactions when refusing sales to intoxicated patrons. Methods to overcome obstacles included observing customers when they arrive; relying on managers, bartenders, and other servers for help; slowing down activities during busy times or slowing service to intoxicated customers; eliminating tips; having support from management; dividing RBS tasks among different staff; serving water to intoxicated customers; and setting limits on the number of drinks served.

All servers saw benefits of continued information, training, and support for RBS methods. The servers also described topics that would be helpful. The perceived benefits of a continuing professional development program included peer support, sense of community, networking among servers, keeping updated on new information, and helping new servers. These included interactive learning activities, prompts, refreshers on laws, IDs, recognizing intoxication, drink counting, and refusing service, instruction on how to deal with minors, forums for sharing stories and tips with other servers, help for newer servers, ways of managing difficult customers and de-escalating conflict, polls, infographics, and reminders. Almost all servers indicated that they would be willing to share their experiences and provide feedback on RBS methods in the *WayToServe Plus* Facebook group.

##### Survey

Table 1 presents the profile of the sample of alcohol servers (n=24) who completed the web survey. Alcohol servers ranged in age from 20 to 40 (mean 28.8, SD 5.4) years; a majority were non-Hispanic White (7/24, 29% Hispanic), and there were slightly more female individuals than male individuals. Most servers worked in restaurants and bars as alcohol servers and bartenders and were a mix of new (7/24, 29% had worked less than 1 year) and experienced (15/24, 63% had worked 3 years or more) servers.

Although most servers were very sure of their ability to apply RBS methods, some encountered problems. A sizable minority were only somewhat sure or unsure that they could verify the validity of IDs (3/24, 12%; mean 4.79 out of 5, SD 0.66), check IDs for age of patron (2/24, 8%; mean 4.83, SD 0.64), count number of drinks to prevent intoxication (6/24, 25%; mean 4.63, SD 0.77), recognize if a patron is intoxicated (6/24, 25%; mean 4.63, SD 0.88), or refuse alcohol service to an intoxicated patron (5/24, 21%; mean 4.75, SD 0.53). The obstacles to RBS methods cited included busy serving environments (10/24, 42%), customer intoxicated before arriving (10/24, 42%), regular customers expecting heavier pours (6/24, 25%), coworker or management pressure to not follow RBS regulations (5/24, 21%), fear of sacrificing tips (3/24, 12%), and checking IDs in a group of customers (2/24, 8%). Servers suggested several ways to overcome these obstacles, such as checking everyone’s ID (11/24, 46%), getting support from management to follow rules (5/24, 21%), taking a moment to breathe in busy environments (9/24, 38%), serving water (6/24, 25%) or food (5/24, 21%) to patrons that need to sober up, monitoring patrons for signs of intoxication (13/24, 54%), and involving a manager in handling difficult customers (13/24, 54%).

Almost all servers felt that the management of their establishment was supportive of RBS methods, agreeing that management believes RBS methods are beneficial (22/24, 92%; mean 4.54 out of 5, SD 0.66) and management takes RBS methods seriously (22/24, 92%; mean 4.67, SD 0.64). However, 21% (5/24) reported that management at their establishment provided support for RBS methods only sometimes, rarely, or never. The most common support was help serving when the establishment gets busy (16/20, 80%), answering questions about RBS methods (17/21, 81%), helping servers refuse service to a customer (14/20, 70%), helping check IDs during busy periods (9/20, 45%), and highlighting things to be on alert for before a shift (9/19, 47%).

Servers favorably evaluated the idea of professional development. Overall, 71% (17/24) of servers expressed interest in receiving ongoing information and activities from *WayToServe* to help keep them up-to-date and be able to use the RBS methods. The benefits servers saw from this professional development for themselves would be receiving tips and tricks from other servers, getting refreshers on everyday work practices, helping other servers who need it, and providing a place to vent about poor experiences while serving alcohol (Table 2). Benefits for the establishment included having servers be on the same page when it comes to serving alcohol and remaining in good standing with the state’s alcohol licensing agency. Finally, 46% (11/24) of the servers said they were somewhat or very likely to join a Facebook group with the professional development content, and 21% (5/24) might join it. The topics of most interest to servers included refreshers on signs of intoxication, unusual or humorous experiences by another server, quizzes that test knowledge of alcohol serving laws, servers sharing positive or negative on-the-job experiences, stories from other servers about how they used an RBS method, and polls on what the servers believe the community thinks about alcohol serving topics (Table 2). Less popular topics were servers sharing experiences via Facebook Live, refreshers on laws and penalties, information on new state laws, refreshers on ID checking, interactive learning activities, and instruction on using RBS methods.

**Table 1:** Profile of alcohol server samples in formative research.

Profile		Server survey (n=24)	Usability testing (n=20)	Field pilot study	
				Prototype group (n=59)	Control group (n=51)
**Type of licensed sales, n/N (%)**					
	On site (by the drink)	—^a^	13/20 (65)	38/59 (64)	39/51 (77)
	Off site (package)	—	1/20 (5)	7/59 (12)	8/51 (16)
	Both	—	6/20 (30)	13/59 (22)	4/51 (8)
**Type of establishment, n/N (%)**					
	Bar	4/24 (17)	1/20 (5)	—	—
	Restaurant	12/24 (50)	14/20 (70)	—	—
	Nightclub	0/24 (0)	2/20 (10)	—	—
	Brewery	3/24 (12)	2/20 (10)	—	—
	Distillery or winery tasting room	0/24 (0)	1/20 (5)	—	—
	Other	5/24 (21)	1/20 (5)	—	—
**Job type, n/N (%)**					
	Bartender	8/24 (33)	7/20 (35)	15/59 (25)	13/51 (26)
	Server	12/24 (50)	9/20 (45)	27/59 (46)	24/51 (47)
	Manager	2/24 (8)	1/20 (5)	7/59 (12)	7/51 (14)
	Other	2/24 (8)	3/20 (15)	9/59 (15)	6/51 (12)
**Years of experience, n/N (%)**					
	<1	7/24 (29)	1/20 (5)	14/59 (24)	6/51 (12)
	1-2	1/24 (4)	1/20 (5)	14/59 (24)	9/51 (18)
	2-5	8/24 (33)	5/20 (25)	10/59 (17)	14/51 (27)
	**>**5	8/24 (33)	13/20 (65)	20/59 (34)	22/51 (43)
Age (y), mean (SD)		28.8 (5.3)	32.2 (4.7)	33.1 (11.5)	34.5 (11.7)
**Race or ethnicity, n/N (%)**					
	African American	0/24 (0)	0/20 (0)	1/51 (2)	0/51 (0)
	American Indian or Alaska Native	1/24 (4)	2/20 (10)	2/51 (4)	3/51 (6)
	Asian	0/24 (0)	0/20 (0)	1/51 (2)	4/51 (8)
	Hispanic	7/24 (29)	8/20 (40)	13/59 (22)	17/51 (33)
	Native Hawaiian or other Pacific Islander	2/24 (8)	0/20 (0)	4/51 (8)	0/51 (0)
	Non-Hispanic White	11/18 (61)	9/20 (45)	27/48 (56)	30/51 (59)
**Sex, n/N (%)**					
	Male	13/24 (54)	12/19 (63)	41/59 (70)	18/48 (38)
	Female	10/24 (42)	7/19 ((37)	17/59 (30)	29/48 (60)
	Other	1/24 (4)	0/19 (0)	0/59 (0)	1/48 (2)

^a^Not available.

**Table 2:** Topics of interest in continuing professional development identified by alcohol servers (n=23).

Topic			Participants, n (%)
**Benefits to servers**			
	Tips and tricks from other servers	18 (78)	
	Refreshers on everyday work practices	13 (56)	
	Providing help to other servers who need it	12 (52)	
	Having a place to vent about poor experiences while serving alcohol	11 (48)	
**Benefits to establishments**			
	Having servers on the same page when it comes to serving alcohol	21 (91)	
	Remaining in good standing with the state’s licensing body	13 (56)	
**Professional development topics of interest**			
	Stories from other servers about how they used an RBS^a^ method	8 (35)	
	Unusual or humorous experience by another server	11 (48)	
	Refreshers on signs of intoxication	12 (52)	
	Quizzes that test knowledge of alcohol serving laws with prizes	9 (39)	
	Servers sharing positive or negative on-the-job experiences	9 (39)	
	Polls on what the server community thinks about alcohol serving topics	8 (35)	
	Interactive activities that help maintain a skill	7 (30)	
	Information on new state laws	7 (30)	
	Question and answer posts	9 (39)	
	Interactive learning activity for applying an RBS method with feedback	6 (26)	
	How to use an RBS method	5 (22)	
	Refreshers on ID checking	5 (22)	
	Refreshers on laws and penalties pertinent to servers	5 (22)	
	Servers sharing their experiences via Facebook Live segments	4 (17)	

^a^RBS: responsible beverage service.

#### Survey on Acceptability, Feasibility, and Usability of WayToServe Plus Prototype

Table 1 presents the profile of the sample of alcohol servers in the usability test of the *WayToServe Plus* prototype (n=20). They were aged 25 to 42 (mean 32.2, SD 4.7) years, and the majority were non-Hispanic White (8/20, 40% were Hispanic) and predominately female individuals. Most worked in on-premises alcohol sales establishments, especially restaurants; however, several worked in nightclubs, breweries, and distillery or winery tasting rooms. The main job types were bartender and alcohol server. Most were experienced alcohol servers, with 90% (18/20) working for >2 years as a server.

Alcohol servers rated the posts in *WayToServe Plus* prototype as highly appropriate for themselves and their establishment, very acceptable, and useful (with average ratings of all social media posts and the video being above the scale midpoint; Table 3). They evaluated posts on management and house policy most favorably (means ranged from 3.80 to 4.30), compared with posts on additional training (means ranged from 3.30 to 3.85) and disruptive customers (means ranged from 3.58 to 4.05). The prototype videos were very favorably evaluated in terms of appropriateness, acceptability, and usefulness (means 3.80-4.25). Of the 20 servers, 8 (40%) rated the prototype as usable on the SUS, and 90% (18/20) evaluated it as user-friendly (good, excellent, or best imaginable).

Most servers indicated that they would use *WayToServe Plus* if it was available. Specifically, 60% (12/20) felt that they would like to use it in the future, and 85% (17/20) were interested in getting ongoing information and activities from *WayToServe Plus* to help keep up-to-date and be able to use RBS methods. When considering specific posts, most servers said they would engage with the posts (view, react to, comment, and share), with the number who would read and react to them being especially high. Slightly fewer servers said they would comment on or share their own posts, but ≥45% (9/20) said they would do so. Videos were the most engaging, with >70% (14/20) saying they would read, react, comment on, and share them (Table 3). In addition, 85% (17/20) of the servers would use an interactive learning activity if posted in the *WayToServe Plus* component.

**Table 3:** Acceptability and potential engagement with messages in the *WayToServe Plus* prototype usability survey (N=20).

		Social media posts by topic				Videos
		Professionalism	Premises management	Advanced responsible beverage service skills		
**Acceptability of posts, mean (SD)**						
	Appropriate for me	3.70 (1.45)	4.20 (0.83)	3.90 (0.85)	4.15 (1.27)	
	Appropriate for establishment	3.85 (1.35)	4.25 (0.91)	3.58 (1.17)	3.80 (1.61)	
	Acceptable to me	3.80 (1.40)	4.30 (0.66)	3.95 (0.89)	4.25 (1.02)	
	Useful to me	3.30 (1.45)	3.80 (0.95)	4.05 (1.05)	4.05 (1.32)	
**Potential engagement with posts, n (%)**						
	Would read post	12 (60)	16 (80)	16 (80)	18 (90)	
	Would react to post	10 (50)	13 (65)	15 (75)	15 (75)	
	Would comment on post	12 (60)	10 (50)	10 (50)	15 (75)	
	Would share post	11 (55)	9 (45)	11 (55)	14 (70)	

#### Field Pilot Test of WayToServe Plus

The profile of the alcohol servers participating in the intervention group (n=59) and control group (n=51) in the pilot test is presented in Table 1. Intervention group participants ranged in age from 18 to 65 (mean 33.1, SD 11.5) years, were mostly non-Hispanic White (13/59, 22% Hispanic), and were predominately female individuals. By job type, most worked in on-premises sales establishments as bartenders or alcohol servers. They were a mix of new and experienced servers (14/59, 24% had worked less than 1 year and 20/59, 34% had worked 5 years or more).

Alcohol servers had high engagement with the *WayToServe Plus* professional development component. Overall, 83% (50/60) viewed at least 1 post, and they viewed an average of 14.85 (SD 12.41) posts over 4 weeks (approximately 24 posts were displayed in any 4-week period). Just under half of the servers (28/60, 47%) reacted (eg, liked) or commented on a post in the *WayToServe Plus* group. Servers on average reacted to 4.17 posts (SD 7.08).

Alcohol servers evaluated the *WayToServe Plus* component as highly usable and its content as appropriate. The mean rating on the SUS scale was 81.10 out of 100, with 88% (52/59) giving it a score of ≥68, a common threshold for usability on this scale. They also gave it high marks on user-friendliness (mean 5.81 out of 7). Many felt that the component (52/59, 88% agreed or strongly agreed; mean 4.42 out of 5, SD 1.19) and its content (50/59, 84%; mean 4.31, SD 1.25) were appropriate for them as alcohol servers and aligned with their establishment’s atmosphere (48/59, 81%; mean 4.12, SD 0.79). Most found the posts (49/59, 83%; mean 4.08, SD 0.75) and other servers’ comments on the posts (47/59, 80%; mean 4.05, SD 0.78) to be useful. A large majority of the alcohol servers said that they were likely to use *WayToServe Plus* in the future (46/59, 78% somewhat likely or very likely; mean 3.95 out of 5, SD 0.99).

Servers in the control group were similar in characteristics to those in the intervention group (Table 1), although control servers had more years of experience on average. Alcohol servers who received the *WayToServe Plus* prototype were compared with those in the control group in their reported self-efficacy and response efficacy for implementing RBS methods as an indicator of the potential impact of the *WayToServe Plus* program. Given the small sample size, we planned a priori to calculate the effect size estimate, *d,* rather than perform a standard statistical significance test. Ratings on self-efficacy were higher among servers in the prototype group (mean 4.53, SD 0.57) than servers in the control group (mean 4.33, SD 0.77; *d*=0.30). Likewise, ratings on response efficacy were greater in the prototype group (mean 4.68, SD 0.68) than in the control group (mean 4.39; *SD* 0.83; *d*=0.38).

### Phase 2

Phase 2 was funded in September 2022. Baseline assessment of licensed alcohol premises (n=179) in California (n=59), New Mexico (n=60), and Washington (n=60) was conducted in 2022-2023 using the pseudopatron protocol, and results are available elsewhere [56]. The recruitment of premises to have servers trained and join the Facebook private group containing the professional development posts is ongoing. Posttest assessment is planned for summer and fall, 2024 with results expected to be published in 2025.

## Discussion

### Principal Findings

The development of a professional development extension of our RBS training course aims to improve the efficacy of RBS training in the field. The formative research confirmed that owners, managers, and alcohol servers considered a professional development component for RBS to be beneficial, and a large majority would be interested in using such a program. Many owners and managers have already taken steps to help servers implement and maintain their RBS skills, and several of them felt that *WayToServe Plus* would complement and aid in these efforts. A previous study found that managers trained in RBS also trained their staff in cutting off intoxicated patrons and handling fake IDs [57]. Alcohol servers considered the *WayToServe Plus* prototype to be highly appropriate, acceptable, usable, and useful. Many servers followed (ie, viewed a post) and engaged (ie, reacted to or commented on a post) with the prototype. These results are consistent with the literature citing education and lifelong learning as essential in the hospitality industry to maintain job competence, be productive, and be valued employees [37]. A formal professional development program, such as *WayToServe Plus*, might help employees improve RBS practice faster than informal training by owners and managers [32]. Continued professional development might also increase employee retention and reduce absenteeism by improving confidence in role, clarifying job expectations, helping to manage stressful situations, and increasing job satisfaction and commitment [29,58].

Formative research identifying the topics of interest to servers likely contributed to creating highly engaging posts in the *WayToServe Plus* prototype. Most servers said that they would be interested in receiving ongoing information and support for RBS methods, and many would enroll in the *WayToServe Plus* component in the future. Moreover, the *WayToServe Plus* prototype appeared to improve theoretic mediators of effective RBS training. Continuing professional development programs should be more effective when personally meaningful to learners [30,59]. Together, these findings suggest that the ongoing professional development in *WayToServe Plus* is likely to improve RBS practices in the upcoming RCT and when disseminated with the *WayToServe* RBS training.

Owners, managers, and servers felt that the professional development content fit with the atmosphere of their licensed establishments. Fit might be further enhanced by providing the information in easily digestible and relatable formats that do not require a large time commitment. Fit is an important innovation characteristic that predicts adoption [39], and continuing professional development programs may be most effective when reflecting the context and experiences of learners [30].

The formative research provided insights into the potentially effective content of a professional development component. Servers and managers wanted skills training on refusing service, handling intoxicated and difficult customers, conflict resolution, communication, drink counting, and recognizing intoxication; serving at special events; ID checking; serving laws and penalties; and prohibited conduct (eg, recreational cannabis, drinking on the job, and firearms). These represent a combination of generic as well as job-specific information and skills for alcohol service, a common combination of skills in the hospitality industry [36,37]. Servers suggested several message features that would promote engagement with the professional development content, including positive messages; relaxed, conversational tone; humor; infographics or charts; articles; videos; questions and answers; resources; reminders; interactive activities; badges or rewards; polls; quizzes; games; weekly discussion topics; tips, stories, and comments from experienced servers; and opportunities to share experiences. Sharing ideas and experiences among servers and creating learning communities where they can work collaboratively should facilitate the success of continuing professional development [59,60]. User-generated content stands out as a key feature of social media platforms and holds significant sway in shaping social norms, particularly through the process of opinion leadership [39]. Managers and servers were interested in developing professionalism, such as understanding the roles of management, building a community in the hospitality environment, and enhancing hospitality careers. Cultivating or enhancing a sense of professionalism among servers could potentially elevate their regard for customer and community safety (ie, fostering professional norms [29]), strengthen their commitment to their roles, and motivate the consistent use of RBS methods.

The findings supported the use of a social media platform to deliver the professional development content. In 2021, most adults used social media (72%), including >80% of those aged 18 to 49 years [61], for information and peer connections that can be influential [62,63]. Web-based learning is common in vocational education and continuing professional development, providing advantages in terms of low cost, time efficiency, media-rich presentations, and interactivity [31,59,60]. Our plan to deliver the professional development content on an ongoing basis should help confer mastery of RBS techniques taught initially in the single, intensive *WayToServe* course by providing time for servers to set goals to improve behavior, assess current performance, and receive timely feedback to make improvements [60]; however, to be effective, servers will need to be self-directed learners with sufficient motivation to engage with the post. Effectiveness and motivation may increase when coupled with in-person instruction and mentoring from managers and experienced servers [31,59,60], rather than replacing this on-the-job support.

We chose to deliver the *WayToServe Plus* over Facebook because (1) the *WayToServe* training had an existing Facebook page with approximately 20,000 followers; (2) despite some decline in its user base [64-66], Facebook still reaches a large majority of adults including more than 70% adults aged 18-49 years by one estimate in 2021 [61]; and (3) Facebook’s private group feature will control treatment presentation to prevent contamination when testing the effectiveness of *WayToServe Plus*. Video content appeared to be especially popular, which was not surprising given the popularity of video-dominated social media such as YouTube and TikTok [61,67]. Theoretically, visual depictions should be effective at teaching skills through observational learning [40]. To broaden the appeal of the *WayToServe Plus* component, some posts should be linked to relevant content posted on Instagram, YouTube, and other highly popular social media. Once disseminated, it may be most effective to deliver *WayToServe Plus* messaging through multiple social media platforms.

### Limitations

The formative research and upcoming RCT evaluating the *WayToServe Plus* professional development component will be limited by conducting them with servers in only 3 states in the western United States, California, New Mexico, and Washington State, where the *WayToServe* is an approved RBS training provider. However, these states are diverse in population size, history of RBS training requirements (ie, New Mexico and Washington State have required RBS for over 2 decades, whereas California’s requirement was new in 2022), and content requirement for RBS training (eg, California requires more content for managers than New Mexico and Washington State). The selection method using clustering of establishments in California and Washington State could introduce a design effect, but it was balanced against cost controls and project feasibility. The findings pertain to web-based professional development content, not other forms of support delivered in person or in print, but web delivery creates a high-quality, high-fidelity, engaging learning environment [68]. Uptake of *WayToServe Plus* will undoubtedly vary among servers and across establishments, which could diminish its effectiveness. However, the formative research suggests that many servers will engage with the professional development posts. The upcoming evaluation of *WayToServe Plus* will be strengthened by random selection of the licensed establishments; random assignment to experimental conditions; observational measures of refusal rates; and blinding of PiP teams, establishment management, and alcohol servers.

### Conclusions

If successful, this study has the potential to improve the effectiveness of evidence-based RBS training and reduce the negative consequences of DWI. The results will also provide evidence that personnel in regulated industries that affect public health and safety, such as hospitality, can be trained to improve compliance with state policies and regulations. Furthermore, it will show whether professional development can be effective for individuals without specialized professional education. Far from *low-skilled*, alcohol service requires key skills in managing emotions, communication, problem-solving, and flexibility [36] as well as learning and applying the regulations and best practices surrounding responsible alcohol service. It should be amenable to improvement through ongoing professional development between state-required retraining in RBS techniques. The market for RBS training is large; therefore, improvements in this common intervention could have a substantial impact on DWI rates. No RBS training provider currently provides ongoing professional development as extensive as is planned for *WayToServe Plus,* so it should be seen as a value-added component for many licensed establishments, improving its dissemination potential.

## Abbreviations

DWI: driving while intoxicated
PiP: pseudointoxicated patron
RBS: responsible beverage service
RCT: randomized controlled trial
SUS: System Usability Scale

## Data Availability

The data set generated and analyzed during the pilot study is available in the Inter-university Consortium for Political and Social Research (ICPSR) repository [69].
